# Expression of Y-box-binding protein dbpC/contrin, a potentially new cancer/testis antigen

**DOI:** 10.1038/sj.bjc.6602987

**Published:** 2006-02-14

**Authors:** Y Kohno, Y Matsuki, A Tanimoto, H Izumi, T Uchiumi, K Kohno, S Shimajiri, Y Sasaguri

**Affiliations:** 1 Department of Pathology and Cell Biology, School of Medicine, University of Occupational and Environmental Health, Kitakyushu, Japan; 2 Department of Ophthalmology, School of Medicine, University of Occupational and Environmental Health, Kitakyushu, Japan; 3 Department of Molecular Biology, School of Medicine, University of Occupational and Environmental Health, Kitakyushu, Japan; 4 Department of Surgical Pathology, Kyushu Koseinenkin Hospital, Kitakyushu, Japan

**Keywords:** dbpC/contrin, human tumours, immunohistochemistry, cancer/testis antigen, cancer stem cells

## Abstract

Y-box-binding proteins are members of the human cold-shock domain protein superfamily, which includes dbpA, dbpB/YB-1, and dbpC/contrin. dbpC/contrin is a germ cell-specific Y-box-binding protein and is suggested to function as a nuclear transcription factor and RNA-binding protein in the cytoplasm. Whereas ubiquitous dbpB/YB-1 expression has been well studied in various types of human carcinomas as a prognostic or predictive marker, the dbpC/contrin expression in human tumour cells has not been reported. In this report, we provide the first evidence showing that dbpC was highly expressed in human testicular seminoma and ovarian dysgerminomas, and in carcinomas in other tissues and that its expression in normal tissues is nearly restricted to germ cells and placental trophoblasts. These results indicate that dbpC/contrin would be a potentially novel cancer/testis antigen.

Y-box-binding proteins are nucleic acid-binding proteins and are members of the human cold-shock domain protein superfamily (CSPs), which includes dbpA, dbpB/YB-1, and dbpC/contrin (Kohno *et al*, 2003). The CSPs are eukaryotic homologues of bacterial cold-shock proteins, which function as RNA chaperones (Matsumoto and Wolffe, 1998). Human heat-shock proteins have been well studied; however, much is not known about the functional roles of cold-shock proteins. In general, the cold-shock response includes growth arrest and a reduction in protein synthesis in bacteria (Jolly and Morimoto, 2000). The eukaryotic Y-box-binding proteins have been not known to be involved in the cold-shock response; however, recent study indicated a block in cell proliferation after low-temperature shift in YB-1 knockout chicken cell line (Matsumoto *et al*, 2005).

The Y-box-binding proteins participate in various reactions, including DNA repair, transcription, and translation (Kohno *et al*, 2003). Of the human Y-box-binding protein family members, dbpB/YB-1 was first described in 1988 as a ubiquitously expressed transcriptional factor that can bind to the CCAAT-box of the major histocompatibility complex (MHC) class II promoter (Didier *et al*, 1988). The dbpA gene was first identified in 1988 (Sakura *et al*, 1988) and cloned in 1995, encoding a protein highly expressed in skeletal muscle (Kudo *et al*, 1995), and that of dbpC/contrin was cloned in 1999 as coding for a testis-specific protein distinct from other Y-box-binding proteins (Tekur *et al*, 1999). The ubiquitous expression of the dbpB/YB-1 gene in human normal and neoplastic tissues has been well studied and found to be closely associated with the expression of p-glycoprotein, the product of the multidrug resistance 1 (MDR1) gene, in breast cancers (Bargou *et al*, 1997), osteosarcomas (Oda *et al*, 1998), ovarian cancers (Kamura *et al*, 1999), and synovial sarcomas (Oda *et al*, 2003). It is also associated with the expression of DNA topoisomerase II and proliferating cell nuclear antigen in colon (Shibao *et al*, 1999) and lung (Gu *et al*, 2001) cancers. Thus, the nuclear localisation of dbpB/YB-1 is significantly related to malignant behaviour, and, consequently, this transcriptional factor is a tumour-biologic factor indicating a poor prognosis (Janz *et al*, 2002).

In contrast, the expression of dbpA was first reported to be restricted to the heart and skeletal muscles (Kudo *et al*, 1995). It functions as a transcriptional repressor of MHC I-A and vascular endothelial growth factor genes (Lloberas *et al*, 1995; Coles *et al*, 2002). A recent report indicated somatic mutations and single-nucleotide pleomorphism in the promoter of dbpA to be associated with hepatocarcinogenesis (Hayashi *et al*, 2002). On the other hand, dbpC/contrin is a germ cell-specific Y-box-binding protein and is suggested to function as a nuclear transcription factor and RNA-binding protein in the cytoplasm (Tekur *et al*, 1999). However, its expression and distribution have not been studied in human neoplastic tissues. In the present study, therefore, we investigated the expression and distribution of the dbpA and dbpC/contrin in human normal and neoplastic tissues by using antibodies raised against C-terminal synthetic peptides of these proteins.

We demonstrated that dbpC, but not dbpA, was highly expressed in human normal spermatogonia/spermatocytes, oocytes, and placental trophoblasts as well as in testicular seminoma, ovarian dysgerminomas, and various histological types of human cancer tissues. Therefore, a possibility is suggested that the dbpC would be a novel cancer/testis (C/T) antigen and also a marker for cancer stem cells.

## MATERIALS AND METHODS

### Preparation of human tissue samples

Normal human tissue samples of general organs were obtained from three autopsy cases and from non-tumour parts of surgically resected specimens (Table 1). For human neoplastic tissue samples, 123 cases of surgically resected tumours including 21 germ cell tumours of ovarian and testicular origin were examined in the Department of Pathology and Cell Biology at University of Occupational and Environmental Health in Kitakyushu, Japan (Tables 2 and 3). These cases were classified according to the World Health Organization Histological Typing of each tissue. The diagnosis was re-evaluated and confirmed by at least three board-certified surgical pathologists who had examined formalin-fixed, paraffin-embedded tissue sections stained with haematoxylin and eosin (H&E) or appropriate immunohistochemical stains.

### Preparation of antibodies against CSPs

Polyclonal antibodies were raised against dbpA and dbpC/contrin by multiple immunisation of New Zealand white rabbits with synthetic peptides. The sequence of the synthetic peptide for dbpA was CGKEAKAGEAPTEN, and that for dbpC/contrin, CAPVNSGDPTTTILE. The specificity of the antibodies was confirmed by Western blotting and immunostain with peptide competition (not shown).

### Immunohistochemistry of tissue samples

Immunohistochemical staining was performed by using the antibody-linked dextran polymer method (Envision, DAKO, Tokyo). Deparaffinised and rehydrated 5-*μ*m sections were antigen-retrieved by heating them in Target retrieval solution (DAKO) for 20 min, and then incubating them in 3% H_2_O_2_ for 10 min to block endogenous peroxidase activity. The sections were thereafter rinsed and incubated with rabbit polyclonal anti-CSPs antibodies. The second antibody/peroxidase-linked polymers were then applied, and the sections were incubated with a solution consisting of 20 mg of 3.3′-diaminobenzidine tetrahydrochloride, 65 mg of sodium azide, and 20 ml of 30% H_2_O_2_ in 100 ml of Tris-HCl (50 mmol l^−1^, pH 7.6). After having been counterstained with Meyer's haematoxylin, the sections were observed under a light microscope. For the CSP immunohistochemistry of normal human tissues, the intensity of the immunohistochemical reaction was graded into four categories: (−), cells with negative staining; (+), cells with a low staining intensity; (++), cells with moderately stronger intensity than (+) but less than (+++); (+++), cells with a marked staining intensity. For the neoplastic tissues, positive areas comprising less than 10% of the neoplasms were considered as negative staining. Positive areas that were equal to or more than 10% were defined as positive staining and were graded into three categories: (+), positive area of 10–30%; (++), 30–80%; (+++), more than 80% positive area.

### Cell cultures

Human seminoma cell lines Tera1 (ATCC HTB-105), NEC8 (JCRB0250), choriocarcinoma cell line NJG (IFO50322), and breast cancer cell line MCF7 cell (ATCC HTB-22) were obtained from American Type Culture Collection (Rockville, MD, USA) and maintained in RPMI1640 medium containing 10% fetal calf serum (ICN, Costa Mesa, CA, USA) at 37°C in an atmosphere of 95% air and 5% CO_2_.

### Immunocytochemistry of seminoma cell line NEC8

Human seminoma NEC8 cells were cultured on coverslips, fixed with 95% acetone for 5 min, and allowed to air dry. The cells were then incubated with anti-dbp antibodies for 1 h at room temperature (RT), washed with PBS, and reacted with fluorescein isothiocyanate-conjugated goat anti-rabbit IgG for 1 h at RT. After having been washed with PBS, the specimens were observed under a Nikon fluorescence microscope.

To investigate the subcellular localisation of the dbpC protein, we transfected human seminoma cells (NEC8) by lipofection (Gibco BRL, Grand Island, NY, USA) with plasmids expressing dbpC fused to green fluorescent protein (GFP-dbpC). The cells were cultured for 24 h after the transfection and then observed under the fluorescence microscope.

### Cell fractionation and Western blotting

Cells (2 × 10^7^) were resuspended in 2 ml of ice-cold 10 mM HEPES-KOH (pH 7.9) containing 10 mmol l^−1^ KCl, 0.1 mmol l^−1^ EDTA, 0.1 mmol l^−1^ EGTA, 1 mmol l^−1^ dithiothreitol, and 0.5 mmol l^−1^ phenylmethylsulphonyl fluoride (PMSF) and incubated on ice for 15 min. The cells were then lysed by the dropwise addition of 0.6% NP-40, and the lysates were centrifuged at 400 **g** for 10 min at 4°C. The supernatant was used as the cytoplasmic fraction, and the nuclear pellet was resuspended in 300 *μ*l of ice-cold 20 mmol l^−1^ HEPES-KOH (pH 7.9) containing 0.4 mol l^−1^ NaCl, 1 mmol l^−1^ EDTA, 1 mmol l^−1^ dithiothreitol, and 1 mmol l^−1^ PMSF and incubated for 15 min on ice with frequent gentle mixing followed by centrifugation for 5 min at 4°C to remove insoluble materials. The resulting supernatant was taken as the nuclear fraction. The proteins in the cytoplasmic and nuclear fractions (100 *μ*g) were separated by 10% SDS–polyacrylamide gel electrophoresis and transferred onto a polyvinylidene fluoride membrane. The membrane was immunoblotted with anti-CSP antibodies (diluted 1 : 3000) for 1 h and then developed by chemiluminescence using the ECL kit (Amersham, Piscataway, NJ, USA).

## RESULTS

### Expression of Y-box-binding proteins in normal human tissues (Figure 1 and Table 1)

**Figure 1:**
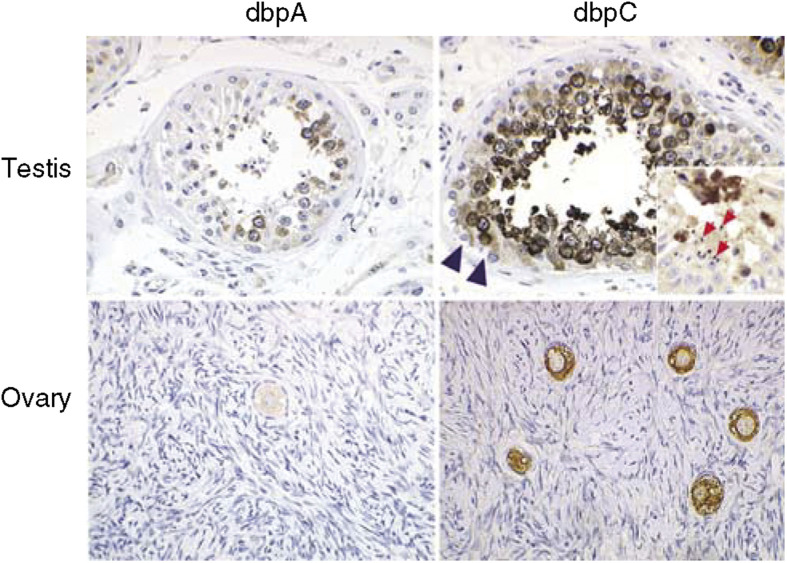
Expression of dbpA and dbpC in normal human testis and ovary. Note the extensive expression of dbpC in the spermatogonia to spermatocytes and in the oocytes. Some cells show a nuclear localisation of the dbpC protein. Sertoli cells (arrowheads) and spermatids (arrows in the inset) are negative for the dbps. On the other hand, the cells located in the inner parts of the seminiferous tubules (spermatocytes) are positive for dbpA.

For detection of dbpA and dbpC expression in normal human tissues, non-tumour areas of surgically resected tumours and tissues from autopsy cases were examined immunohistochemically. The dbpA protein was predominantly distributed in the cardiomyocytes and striated muscle cells (not shown). In the ovary and testis, dbpA was expressed in the spermatocytes located in the inner area of the seminiferous tubules but not in oocytes (Figure 1). The dbpC/contrin protein was markedly expressed in oocytes and testicular germ cells in the stage of spermatogonia to spermatocyte, some of which showed nuclear localization of the protein. The vascular smooth muscle cells in the pulmonary artery, myocardium, and skeletal muscle were also (+) positive for dbpC (not shown), but epithelial cells in respiratory, gastrointestinal, and urogenital tracts were negative. Placental trophoblasts were also positive for dbpC (not shown). The results on the immunolocalisation of these dbps in normal human tissues are summarised in Table 1.

### Expression of dbpA and dbpC in human carcinomas in various organs (Figure 2 and Table 2)

**Figure 2:**
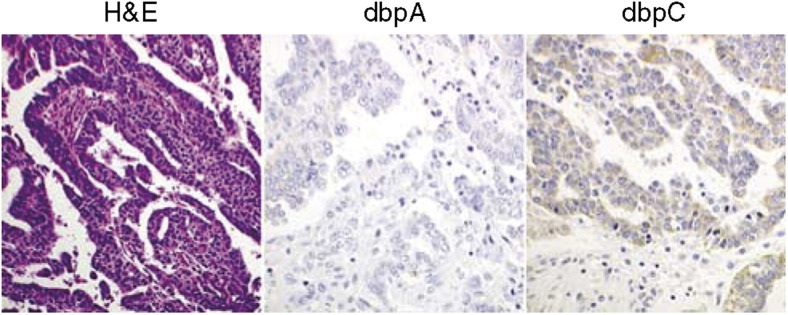
Expression of dbpA and dbpC in a human ovarian adenocarcinoma. The carcinoma cells are weakly positive for dbpC, but negative for dbpA.

As the expression of dbpB/YB-1 has been well studied in many types of human carcinoma cells (Bargou *et al*, 1997; Oda *et al*, 1998; Kamura *et al*, 1999; Shibao *et al*, 1999; Gu *et al*, 2001; Oda *et al*, 2003), we focused on the expression of dbpA and dbpC in human tumour cells in this report. We collected the cancer tissues of the alimentary and respiratory tracts, breast, kidney, prostate, and ovary (10 cases each). As a result, no dbpA was detected in these cancer cells, except for two rare cases of cholangiocellular carcinoma, one renal cell carcinoma, and one ovarian serous cystadenocarcinoma, in which the degree of positive staining was (+) or (++). In contrast, dbpC was expressed in many types of carcinoma cells at a relatively higher frequency than for dbpA; however, all staining was (+) or (++). The frequency of dbpC-positive tumours varied among the histological types. Hepatocellular carcinomas and lung adenocarcinomas were negative in all 10 cases, whereas adenocarcinomas of the colon and cholangiocellular carcinomas exhibited higher frequencies for expressing dbpC (nine out of 10 and eight out of 10, respectively). In total, approximately 40% (46 out of 110) of the carcinomas were positive for dbpC. Even in the negative cases (less than 10% positive cells), however, scattered individual cells were positive for dbpC in the most carcinoma tissues examined (not shown). Figure 2 shows a representative case of dbpC-positive ovarian serous cystadenocarcinoma. The results for all carcinomas examined are summarised in Table 2.

### Expression of dbpA and dbpC in human germ cell tumours (Figure 3 and Table 3)

**Figure 3:**
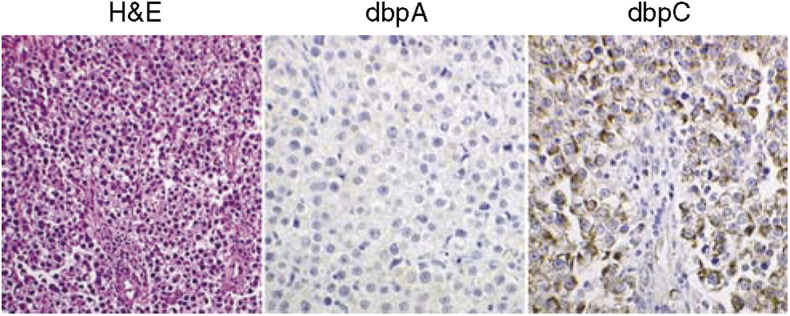
Expression of dbpA and dbpC in human testicular seminoma. This H&E section demonstrates a typical feature of testicular seminoma. The dbpC protein, but not dbpA, is intensely expressed in the cytoplasm of the tumour cells.

All the testicular seminomas and ovarian dysgerminomas tested (cases 1–13), as well as seminoma components in testicular mixed germ cell tumours (cases 14 and 15), showed strong (+++) granular cytoplasmic staining with anti-dbpC antibody, whereas they were negative for dbpA (Figure 3). In other types of germ cell tumours, for example, the tumour cells of yolk sac tumours (cases 14–21) and embryonal carcinoma (case 14), positive immunoreactions for dbpC were focal; however, the cells of choriocarcinomas and of mature or immature teratomas were negative. The results of immunohistochemistry for dbpA and dbpC in the germ cell tumours are summarised in Table 3.

### Analysis of dbpC expression in human seminoma cell lines

**Figure 4:**
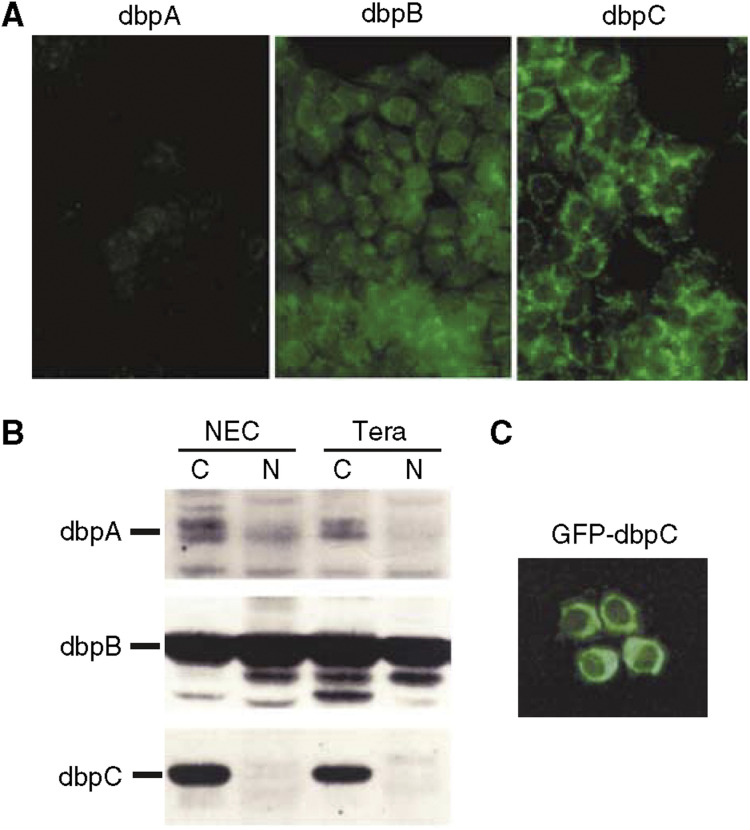
Expression of dbpC in cultured human seminoma cells. (**A**) Demonstration of dbpA, dbpB, and dbpC immunofluorescence in cultured seminoma NEC8 cells. The dbpC is localised in the cytoplasm of the NEC8 cells, whereas dbpA and dbpB expression is weak. (**B**) Western blotting analysis of dbp expression. dbpC is expressed in the cytoplasmic fraction (C) but not in the nuclear fraction (N) from human seminoma NEC8 or Tera cells. dbpB is expressed in both cytoplasmic (C) and nuclear (N) fractions. The expression of dbpA is weakly detected in the cytoplasm or nuclei. (**C**) Expression of GFP-dbpC in NEC8 cells. The green fluorescence indicating the protein is predominantly located in the cytoplasm.

The immunofluorescence staining of the seminoma cell lines showed marked expression of dbpC in the cytoplasm, but only weak expression for dbpA and dbpB (NEC8 in Figure 4A). Western blotting analysis showed cytoplasmic distribution of dbpC protein in both seminoma cell lines examined (NEC8 and Tera), whereas dbpB was localised in both cytoplasmic and nuclear fractions (Figure 4B). When NEC8 cells were transfected with plasmids expressing the GFP-dbpC fusion protein, the green fluorescence was mainly localised in the cytoplasm, indicating a predominant localisation of dbpC even in the condition of overexpression (Figure 4C).

## DISCUSSION

The expression of many genes is regulated at the level of transcription during embryogenesis and spermatogenesis. Translational control, however, represents a major mechanism of gene regulation in germ cell differentiation and early embryogenesis and in late spermatogenesis (Ritcher, 1993; Schafer *et al*, 1995; Hecht, 1998). Germ cells store masked mRNAs, and the unmasking of the mRNAs for protein synthesis plays an important role in the meiotic progression of oocytes to eggs and in the late stage of spermatogenesis (Hecht, 1995; Eddy and O'Brien, 1997). This translational control is likely to be regulated by a number of RNA-binding proteins (Cooke and Elliott, 1997). dbpC, a mammalian homologue of the *Xenopus* germ cell-specific Y-box protein, may serve a similar function, as it is present exclusively in postmeiotic round spermatids in rodent testis and in diplotene-stage and mature oocytes (Gu *et al*, 1999; Tekur *et al*, 1999). Recently, growing numbers of genes that are expressed in male germ cells and malignancies have been designated as CT genes, by which a common functional pathway between spermatogenesis and tumorigenesis (e.g. progression of meiosis and polyploidy, respectively) would be explainable (Old, 2001; Simpson *et al*, 2005). Therefore, the study of the expression of dbpC in malignancies might provide clues for understanding the shared characters between spermatogenesis and tumorigenesis.

Here, we demonstrated for the first time that dbpC is highly expressed in human normal germ cells and trophoblasts, but rarely in no-germline normal tissues, as well as testicular seminomas, ovarian dysgerminomas, and many cancer cells in other organs.

### Expression of dbps in normal and neoplastic tissues

The present study showed unique distribution of the dbpA and dbpC proteins in normal human tissues. dbpA was distributed in the heart, skeletal muscle, and decidual cells, whereas dbpC was predominantly localised in testicular and ovarian germ cells, and in trophoblasts (Table 1). In the human testis, dbpC was expressed intensely in the cells undergoing transition from spermatogonia to spermatocytes. In the neoplastic tissue, dbpA was detected in only a few carcinoma cases among more than 100 cancer cases. In contrast, dbpC was extensively expressed in the seminomas and dysgerminomas. In other organs, many types of cancers were also positive for dbpC (Tables 2 and 3). Thus, dbpC showed restricted expression in normal tissues but a wide distribution in malignancies. This contrasts well with the ubiquitous expression of dbpB in normal and neoplastic cells (Kohno *et al*, 2003).

### dbpC as a potential cancer/testis antigen

Antigens that show expression restricted to normal testicular germ cells, trophoblasts, and malignancies are designated as CT antigens (Old, 2001; Scanlan *et al*, 2004; Kalejs and Erenpreisa, 2005; Simpson *et al*, 2005). As features in germ cells corresponding to those in cancer cells include immortalisation to transformation, meiosis to aneuploidy, and migration to metastasis, it is thought that genetic alteration in cancer cells is the result of the activation of normally silent germline genes. The CT genes support the theory that the aberrant expression of germline genes in cancer cells reflects the reactivation of the silenced gametogenic programmes in terms of tumorigenesis (Simpson *et al*, 2005). At present, 44 CT antigen families are known to be expressed in testicular spermatogonia, spermatocytes, or spermatids and in malignancies of various histological types including lung, gastrointestinal, urogenital cancers (Jungbluth *et al*, 2000, 2002; Scanlan *et al*, 2004; Kalejs and Erenpreisa, 2005). The biological function of CT antigens in either germ cells or cancer cells is not yet clearly understood; however, some of the CT antigens are suggested to be translational or transcriptional regulatory factors or RNA-binding proteins (Simpson *et al*, 2005).

On the other hand, the dbpC protein was initially found as a product of mouse testicular cDNA and shown to be a mammalian homologue of *Xenopus* germ cell-specific nucleic acid-binding protein FRGY2 (Gu *et al*, 1999). It is expressed in mouse round spermatids and mature oocytes and is known to interact with both DNA and RNA. In the present study, we also demonstrated the expression of dbpC to be predominantly in human male germ cells and placental trophoblasts as well as in various malignancies. These features of the tissue distribution and nucleic acid-binding capacity in relation to spermatogenesis are very similar to those of other CT antigens. Although dbpC was also expressed in non-germ cells (myocardium, skeletal muscle, vascular smooth muscle cells in the lung, and adrenal cortical cells), its distribution was more restricted than that of dbpB, which is ubiquitously expressed in normal human tissues (Kohno *et al*, 2003). Thus, taken together, our results indicate that the dbpC would be potentially a novel CT-associated antigen.

As the expression of the CT antigens is detected in spermatogonia or spermatogonial stem cells, the CT antigens could be stem cell markers for tumour cells (Simpson *et al*, 2005). In fact, immunohistochemical analysis has shown that CT antigens such as MAGE-1, MAGE-3, NY-ESO-1, and CT7 are frequently found in only a small proportion of cancer tissues (Jungbluth *et al*, 2000, 2002), an indication that the cancers contain both stem cells and differentiated cells (Simpson *et al*, 2005). In terms of human testicular tumours, many CT antigens including MAGE, GAGE, PAGE-1, SSX2, NY-ESO-1, LAGE-1, and SCP-1 are also demonstrated to have higher frequency of expression in seminomas than in non-seminomas (Yuasa *et al*, 2001). Therefore, these results suggest that less-differentiated seminomas would contain more CT antigen-positive stem cells than any other germ cell tumour or cancer. In the present study, the seminomas and dysgerminomas showed a diffuse and intense expression with the highest frequency (100%) for dbpC among the tumours tested including other non-seminoma germ cell tumours and carcinomas. Even in the carcinomas classified as negative for dbpC because of less than 10% positive cells, scattered dbpC-positive cells were detected (not shown). dbpC would thus be not only a CT antigen but also a marker of cancer stem cells.

### Subcellular localisation of dbpC in seminoma cells

The Western blotting analysis demonstrated that the dbpC protein was detected in the cytoplasmic fraction of seminoma cell lines (Figure 4B), and the cytoplasmic localisation of dbpC was confirmed by exogenous overexpression of GFP-dbpC protein in the NEC8 cells (Figure 4C). These findings indicate that dbpC might function mainly in the cytoplasm and be related to the stability of mRNA and translational regulation in seminoma cells (Gu *et al*, 1999).

In summary, we demonstrated that dbpC was dominantly expressed in normal germ cells and in various malignancies. As the distribution of this protein is very similar to that of CT antigens, we suggest dbpC to be a novel CT antigen and a marker for cancer stem cells.

## Figures and Tables

**Table 1:** Distribution of dbps in human normal tissue

	**dbpA**	**dbpC**
Ovary		
Oocyte	−	+++
Granulosa cell	−	−
		
Testis		
Spermatogonia and spermatocyte	+	+++
Sertoli cell	−	−
Leydig cell	+	−
		
Uterus	−	−
Fallopian tube	−	−
Oesophagus	+	−
Stomach	+	−
Small intestine	−	−
Large intestine	−	−
Liver	−	−
Pancreas	−	−
		
Lung – arterial smooth muscle	−	+
Heart	++	+
Skeletal muscle	++	+
Aorta	−	−
		
Renal tubules	−	−
Bladder	−	−
Spleen	−	−
Bone marrow – megakaryocytes	−	−
Thymus	−	−
		
Brain	−	−
Spinal cord	−	−
		
Thyroid gland	−	−
Adrenal gland	−	−
Cortex – reticular zone	+	+
		
Decidual cell	++	−
Trophoblast	−	++

**Table 2:** Expression of dbpA and dbpC in cancer tissues

			**dbpA**			**dbpC**		
**Organ**	**Histology**	**Cases**	+	**++**	**+++**	+	**++**	**+++**
Ovary	Serous adenocarcinoma	10	1	0	0	2	0	0
Stomach	Adenocarcinoma	10	0	0	0	2	3	0
Colon	Adenocarcinoma	10	0	0	0	2	7	0
Liver	Hepatocellular carcinoma	10	0	0	0	0	0	0
	Cholangiocellular carcinoma	10	1	1	0	2	6	0
Breast	Invasive ductal carcinoma	10	0	0	0	2	3	0
Pancreas	Adenocarcinoma	10	0	0	0	4	2	0
Kidney	Clear cell carcinoma	10	1	0	0	1	0	0
Prostate	Adenocarcinoma	10	0	0	0	4	0	0
Lung	Adenocarcinoma	10	0	0	0	0	0	0
	Squamous cell carcinoma	10	0	0	0	3	3	0
								
	Total cases	110	3	1	0	22	24	0
								
Testis	Seminoma	10	0	0	0	0	0	10
Ovary	Dysgerminoma	3	0	0	0	0	0	3
								
	Total cases	13	0	0	0	0	0	13

**Table 3:** Expression of dbps in human germ cell tumours

**Case**	**Age**	**Sex**	**Primary site**	**Histological type**	**dbpA**	**dbpC**
1	12	F	Ovary	Dysgerminoma	−	+++
2	34	F	Ovary	Dysgerminoma	−	+++
3	27	F	Ovary	Dysgerminoma	−	+++
4	43	M	Testis	Seminoma	−	+++
5	53	M	Testis	Seminoma	−	+++
6	40	M	Testis	Seminoma	−	+++
7	28	M	Testis	Seminoma	−	+++
8	50	M	Testis	Seminoma	−	+++
9	38	M	Testis	Seminoma	−	+++
10	44	M	Testis	Seminoma	−	+++
11	43	M	Testis	Seminoma	−	+++
12	36	M	Testis	Seminoma	−	+++
13	25	M	Testis	Seminoma	−	+++
						
14	33	M	Testis	Seminoma	−	+++
				Yolk sac tumour	−	+
				Embryonal carcinoma	−	+
						
15	37	M	Testis	Seminoma	−	+++
				Yolk sac tumour	−	+
				Embryonal carcinoma	−	−
				Immature teratoma	−	−
						
16	31	F	Ovary	Yolk sac tumour	−	−
						
17	28	M	Testis	Yolk sac tumour	−	+
				Choriocarcinoma	−	−
				Immature teratoma	−	−
						
18	39	F	Ovary	Choriocarcinoma	−	−
						
19	31	M	Testis	Yolk sac tumour	−	+
				Choriocarcinoma	−	−
						
20	43	M	Testis	Yolk sac tumour	−	+
				Embryonal carcinoma	−	−
				Immature teratoma	−	−
						
21	28	M	Testis	Yolk sac tumour	−	+
				Mature teratoma	−	−
